# Progress in Surgery

**Published:** 1899-03-04

**Authors:** 


### Progress in Surgery.

### RHINOLOGY.

(*Continued from page* 367.)

**Neuroses due to Adenoids.**—Hitchcock[20] reported to the American Laryngological Association a case of pronounced epilepsy in which adenoid growth was manifestly an exciting cause of the convulsions. A boy, aged eleven, without hereditary history of neuroses, who was a mouth breather from infancy, had epileptic attacks in the more violent of its forms commencing after an attack of pneumonia at two years, the seizures occurring at intervals of about two to seven weeks. At seven years *petit mal* became superadded, following, as it was said, removal of the tonsils, and kept getting worse until the attacks reached ten a day. When about nine years of age he was placed under treatment by a prominent neurologist, who, observing no improvement from the use of bromides, referred the patient for nasal examination. Marked lymphoid hypertrophy was found and removed under ether, with the result that attacks of all kinds immediately ceased throughout an unbroken period of eighteen months, although just one month after the operation he had to be operated upon for appendicitis, and three months later he had a serious attack of pneumonia. Then the attacks having begun to return in August, 1895, another operation was performed for the removal of remnants of the lymphoid masses in October; and the epileptic attacks again ceased completely for five months. Then a blow on the head was followed by a re-establishment of the *petit mal*, which continued in spite of various forms of treatment.

Another case in which asthma was caused by adenoids is recorded by G. F. Parker.[21] A severe attack of asthma was brought on by an application of cocaine to the nasopharynx, the patient being an asthmatic. Adenoids were found, and after removal of these for deafness, the asthmatic symptoms disappeared.

**Frontal Sinusitis.**—A case of Dundas Grant[22] is recorded, in which the Ogston-Luc operation was performed. A free opening, thorough curettement, the insertion of an india-rubber drainage tube through the infundibulum, and immediate closure of the operation wound by suture of the periosteum, and then of the superficial parts, constitutes the operation. Subsequently a glass syringe was applied to the extremity of the drainage tube, which protruded from the nostril, and an extremely minute quantity of pus was withdrawn. This was repeated daily, and at the end of a week a fine intra-tympanic tube was pushed up through the drain, and the sinus was washed out with boracic acid, pressure being exercised over the wound during the process, a precaution also adopted whenever the patient wished to blow his nose. This was repeated on three successive days. The drainage tube was extracted on the tenth day, and the patient was allowed to return home. There was complete recovery, and no subsequent deformity.

**Maxillary Sinusitis.**—The spontaneous discharge of a foreign body of large size from the maxillary sinus has been reported by Ripault,[23] of Dijon. Puncture through the alveolar process was resorted to for disease attacking the maxillary sinus. The perforation was closed by a tube No. 15. Injections are kept up twice a day by the patient himself. Three months after, the tube having become loose, was forced in the perforation too far, and could not be withdrawn. All attempts at extraction proved unsuccessful. The tube disappeared within the sinus, and I had to be content with inserting another tube larger, and continuing the injection. Three weeks after tube No. 15 was expelled through the nose in the course of an injection. Watson Williams[24] records a somewhat similar case, in which the vulcanite peg used to close the alveolar puncture had been allowed to slip into the antrum. The peg was washed out by the natural antral ostium in cleansing the cavity. D'Arcy Power[25] records a case of empyema of the antrum in a child aged eight years.

**Sphenoidal Sinusitis.**—Schech, in a report on caries of the sphenoid, remarks that while chronic suppurative catarrh of the sphenoidal sinus—vulgo empyæma—frequently runs a latent course or is accompanied with headache, giddiness, or a purulent discharge, empyema combined with diffuse bone disease usually causes severe, dangerous, or fatal results. Owing to its proximity to important osseous fissures, blindness, ocular paralysis, erosion of the carotid, or other vessels, thrombosis, meningitis, sub-dural or brain abscess may be caused. Extensive caries of the sphenoid is usually due to some dyscrasia, *e.g.*, syphilis, malignant growth. He has seen three cases. (1) A woman, aged 28, complained of headache and nasal obstruction, and a tumour, microscopically benign, growing from the lower anterior wall of the sphenoidal sinus, was removed. But there were indications of serious disease. Thus, there was present paralysis of the motor oculi and abducens, while the opticus was intact. As there was purulent discharge from the sinus it was washed out. This was followed by unconsciousness, rigor, fever, and excessive polyuria with a large amount of sugar. The sugar disappeared after a few days. After four weeks ptosis of the left eye and diminution of the visual field occurred, headache increased, with a blood-tinged fœtid discharge from the nose. Two months later there was total blindness; the opening into the sinus was enlarged from the nose. Antispecific treatment had no effect, so the diagnosis of a malignant growth was made. Post-mortem showed a glio-sarcoma. (2) The second and third cases were syphilitic. Both had purulent discharge from the sphenoidal sinus, with bare bone on the posterior superior part of the septum and around the opening of the sphenoidal sinus. Treatment was antispecific and locally regular cleansing of the nasal fossæ, introduction of a probe coated with hexamethylviolet into the sphenoidal sinus and insufflation of iodoform on the carious spots. In spite of treatment the disease lasted for months. One of the patients suffered from an apoplectic attack, with unilateral paralysis of tongue, face, as well as great weakness of the arms and legs. This disappeared in a few months with innunctions of mercury and iodide internally. The other case suffered early from headaches, and, later, from tingling and formication in the arms and finger tips, as well as attacks of vomiting and unconsciousness. Recovery also followed in this case.

